# Quantitative Analysis of Instrument Motion Paths in Cataract Surgery across a Resident’s Training

**DOI:** 10.1016/j.xops.2025.101014

**Published:** 2025-11-26

**Authors:** David Mikhail, Shuting Xie, Michael Balas, Jason M. Kwok, Ana Miguel, Amrit Rai, Amandeep Rai, Peter J. Kertes, Iqbal Ike K. Ahmed, Matthew B. Schlenker

**Affiliations:** 1 Temerty Faculty of Medicine, University of Toronto, Toronto, Ontario, Canada; 2 Department of Computer Science, University of Toronto, Toronto, Ontario, Canada; 3 Department of Ophthalmology and Vision Sciences, University of Toronto, Toronto, Ontario, Canada; 4 Department of Ophthalmology, Private Hospital of La Baie, Avranches, France; 5 Department of Ophthalmology, Central University Hospital of Caen, Caen, France; 6 Prism Eye Institute, Mississauga, Ontario, Canada; 7 John and Liz Tory Eye Centre, Sunnybrook Health Sciences Centre, Toronto, Ontario, Canada; 8 John A. Moran Eye Center, Department of Ophthalmology and Visual Sciences, University of Utah, Salt Lake City, Utah

**Keywords:** Artificial intelligence, Cataract surgery, Computer vision, Motion tracking

## Abstract

**Purpose:**

To objectively quantify the motion paths of surgical instruments during cataract surgery across a resident’s training, identifying patterns of skill acquisition and proficiency development.

**Design:**

An *n* = 1 panel study.

**Subjects:**

One ophthalmology resident performing cataract surgery.

**Methods:**

One hundred cataract surgery videos performed by a single resident from their sixth to 760th case were collected. Advanced motion tracking software (Computer Vision Annotation Tool) was utilized to annotate and track the trajectories of 11 surgical instruments on a frame-by-frame basis. Monotonic trends were assessed using the Mann–Kendall test and Theil–Sen slope estimation, with Spearman correlation measuring the association between case number and performance metric values. Pettitt change-point analysis identified significant transitions in the resident’s skill progression.

**Main Outcome Measures:**

Six key motion parameters, including total path length, average velocity, average acceleration, root mean square jerk, average angular change, and workspace coverage, were extracted for each instrument in each video.

**Results:**

All 11 instruments demonstrated statistically significant reductions in ≥1 motion parameter. Path length consistently decreased across training, with the largest reductions seen in the cannula (–11.8%; 95% confidence interval [CI], –17.4% to –6.8%; *P* < 0.001), phacoemulsification handpiece (–11.5%; 95% CI, –14.1% to –8.7%; *P* < 0.001), and cystotome (–8.9%; 95% CI, –11.8% to –5.9%; *P* < 0.001). The intraocular lens inserter showed the greatest reduction in average angular change of 3.0% (–1.70°) (95% CI, –3.9% to –2.0%; *P* < 0.001). Pettitt analysis demonstrated significant shifts in surgical efficiency at around case 300 for most instruments, although improvements in certain advanced tasks (e.g., lens implantation) emerged later.

**Conclusions:**

This large-scale, frame-by-frame motion tracking study revealed distinct instrument- and task-specific learning curves in cataract surgery, highlighting progressive changes in motion metrics over time. A significant shift at approximately case 300 marked a milestone in the resident’s instrument use patterns. These findings underscore the potential of objective, video-based motion tracking analytics to provide data-driven resident feedback, guiding targeted instruction and standardizing cataract surgery training.

**Financial Disclosure(s):**

Proprietary or commercial disclosure may be found in the Footnotes and Disclosures at the end of this article.

As the population ages, >50 million Americans are projected to develop cataracts by 2050.^1^ To meet this demand, there is a pressing need for competent ophthalmologists and improved tools to efficiently and objectively train them. Effective cataract surgery training is essential to prepare resident ophthalmologists for independent practice, ensuring patient safety and improved visual outcomes. Surgical competency is traditionally assessed through subjective preceptor feedback and case outcomes, both of which lack standardization. Numerous studies have highlighted the need for improved cataract surgery training for ophthalmology residents.^2^, ^3^, ^4^, ^5^, ^6^, ^7^ In the United States, the Accreditation Council for Graduate Medical Education mandates a minimum of 86 cataract surgeries as the primary surgeon during residency; however, these requirements lack uniform metrics for evaluating surgical competence.^8^^,^^9^ In response, several assessment tools have been developed to provide structured feedback and measure residents’ performance in cataract surgery.^9^, ^10^, ^11^, ^12^, ^13^ However, global inconsistencies in training curricula, unvalidated methodologies, reliance on subjective raters, and the time- and labor-intensive demands of training evaluators and implementing these scales have hindered widespread adoption.^14^^,^^15^

One approach to developing objective assessment tools involves the creation of a standardized set of data-driven metrics that can accurately and precisely quantify skill level.^16^ Prior studies have shown that experienced surgeons have more quick, efficient, and precise movements than newer surgeons; thus, motion tracking measurements of metrics such as path length, direction, and speed may enable objective determination of surgical skill.^17^, ^18^, ^19^, ^20^, ^21^ The objective of this study is to track the motion paths of surgical instruments used in cataract surgeries performed by a resident ophthalmologist throughout their training, with the goal of deriving quantitative insights into the cataract surgery learning curve.

## Methods

### Data Source

We retrospectively gathered 100 high-definition video recordings of cataract surgeries performed by a single ophthalmology resident during their first year of surgical training from October 5, 2020 to November 3, 2021. The video recordings spanned from the resident’s 6th to 760th case, all of which were performed independently. The choice of a single resident enables longitudinal analysis of skill evolution and consistency in surgical environment, technique, and learning exposure. These videos were recorded in the operating rooms of the Kensington Eye Institute (KEI), University of Toronto, Ontario, Canada. In the 5-year ophthalmology residency program at the University of Toronto, cataract surgical training begins in the fourth year with a dedicated longitudinal rotation at the KEI. During this period, residents typically perform several hundred phacoemulsification cases under the supervision of multiple attending surgeons. Consequently, the cases examined in this study reflect a single resident’s intensive fourth-year training experience rather than a more gradual, cumulative exposure typical of many US residency programs. A total of 28 different faculty surgeons supervised the resident’s surgeries throughout all recordings. Only complete videos of sufficient quality were included, ensuring criteria such as proper eye centering, absence of motion artifacts, adequate lighting conditions, and unobstructed views free from interference by the surgeon’s hands or other instruments. Additionally, videos with any supervisor intervention were excluded. The videos were recorded at 15 frames per second. Each recording was manually trimmed to begin just before the first surgical step and conclude immediately after the final action. In total, 20.7 hours of video footage were collected, comprising 4.5 million frames at a resolution of 1080 × 1920 pixels.

Our study was approved by the KEI, Trillium Health Partners, and University of Toronto Institutional Review Boards (RIS protocol number: 41734), and adhered to the tenets of the Declaration of Helsinki. Each video was originally recorded for training purposes and did not contain any identifiable patient features or data. Patients undergoing surgery at the KEI provided consent for all video recordings.

### Instrument Motion Tracking

We used advanced motion tracking software (Computer Vision Annotation Tool version 2.2) to annotate each video.^22^ We tracked the motion of the following 11 surgical instruments frame-by-frame: (1) sideport knife; (2) forceps; (3) cannula; (4) keratome; (5) cystotome; (6) phacoemulsification (phaco) handpiece; (7) chopper; (8) irrigation/aspiration (IA) handpiece; (9) lens injector for intraocular lens (IOL) delivery; (10) IOL inserter (e.g., Sinskey hook) for positioning; and (11) Weck-Cel sponge. These categories were not subclassified based on technique (e.g., horizontal versus vertical chopping) or task. For example, the forceps and cannula classes included all visible usage of those instruments on video, regardless of their role in a given surgical phase.

We employed the TrackerMIL model from the Open Source Computer Vision Library (OpenCV) available on the Computer Vision Annotation Tool interface.^23^ TrackerMIL is a tracking algorithm based on multiple instance learning, specifically designed to address challenges such as occlusion, scale variation, and unreliable object appearance features. Utilizing a probabilistic framework, TrackerMIL refines object localization by iteratively leveraging positive and negative samples, providing robustness to partial occlusions and appearance changes.^24^ Semiautomated tracking of instrument tips across frames was monitored and manually corrected by a trained annotator (D.M.) as required to ensure consistent and accurate object localization. Instrument tracking was paused if the instrument left the field of view or if its tip could not be reliably localized in a given frame (e.g., a chopper obscured behind a dense cataract nucleus). Video brightness and contrast settings were manipulated as needed to more clearly delineate instrument tips. Tracking resumed once an instrument returned to view.

### Motion Path Parameters

To quantitatively assess the motion of surgical instruments, we calculated a set of metrics from our frame-by-frame tracking data. Detailed descriptions and formulas for each parameter are available in Appendix S1 (available at www.ophthalmologyscience.org). Briefly, total path length represents the cumulative distance that the instrument tip traveled throughout the entirety of the recording. Angular change quantifies the total magnitude of directional adjustments made by the instrument tip during its motion, calculated as the sum of angles formed between consecutive displacement vectors. Velocity measures how quickly the instrument tip moved from one point to another. Acceleration captures how the speed of the instrument tip changed, representing the rate at which velocity increased or decreased over time. Jerk describes how smoothly the instrument tip accelerated or decelerated by measuring the rate at which acceleration changed over time. Workspace coverage refers to the spatial area traversed by the instrument within the video frame.

### Statistical Analysis

Aggregated parameter values spanning the duration of the instrument’s usage in a video were calculated to enable analyses between cases. These parameters included total path length, average velocity, average acceleration, root mean square (RMS) jerk, average angular change, and total workspace coverage. Scatterplots with nonlinear trend lines were generated for each aggregated parameter and instrument combination, using the locally estimated scatterplot smoothing regression method.^25^

To detect the presence of a monotonic trend, we applied the nonparametric Mann–Kendall trend test for the motion path parameters for each instrument over the resident’s training.^26^^,^^27^ To quantify the magnitude of identified trends, the slope and corresponding confidence intervals (CIs) were calculated using the Theil–Sen estimator (also known as Sen slope).^28^^,^^29^ This nonparametric approach estimates linear trends by selecting the median slope from a series of lines generated through all pairs of data points. Sen slope is particularly robust, as it minimizes the influence of extreme values and maintains reliability even in the presence of outliers or nonnormally distributed data. Percentage changes were calculated to standardize and interpret the rate of change of each parameter relative to its typical value, which is expressed as a function of pixels. Specifically, the percentage change per surgical case was obtained by dividing the Sen slope estimate by the median value of the parameter and multiplying by 100. This approach expresses the rate of change as a proportion of the central value of the parameter, facilitating comparisons across parameters with varying scales or units. Similarly, the 95% CIs for the percentage change were calculated by dividing the lower and upper bounds of the Sen slope CI by the median value and expressing these as percentages. The percentage change in motion path parameters was calculated for all instruments per every 10 videos, corresponding to approximately 75 consecutive cases on average.

To further investigate the relationship between case number and motion path parameters, Spearman rank correlation coefficient was calculated for each instrument and parameter.^30^ Whereas Sen slope quantifies the rate of change, Spearman correlation measures the strength and direction of the association between case number and motion path parameters. This provides additional insight into whether improvements occur consistently across training. For example, substantial plateaus or fluctuations in the learning curve may weaken the correlation, even if the overall trend indicates significant improvement. In contrast, a high correlation would suggest that improvement follows a smooth and consistent trajectory.

To identify specific points during training where significant shifts in motion path trends (change-points) occurred, we applied Pettitt method. This nonparametric, rank-based technique uses the Mann–Whitney *U* statistic to compare data distributions before and after a potential change-point, identifying the data point with the largest difference as the change-point.^31^ Change-points may therefore represent transitions in surgical proficiency. This analysis was conducted for total path length and average angular change for each instrument to provide insights into how surgical skills evolved during training.

All statistical analyses were performed using R version 4.4.2 (R Foundation for Statistical Computing) with a prespecified, 2-tailed significance level of *P* = 0.05. Given that multiple comparisons were performed (for each instrument and motion path parameter), the Bonferroni correction method was applied to all statistical analyses.^32^

## Results

### Baseline Characteristics

**Figure 1:**
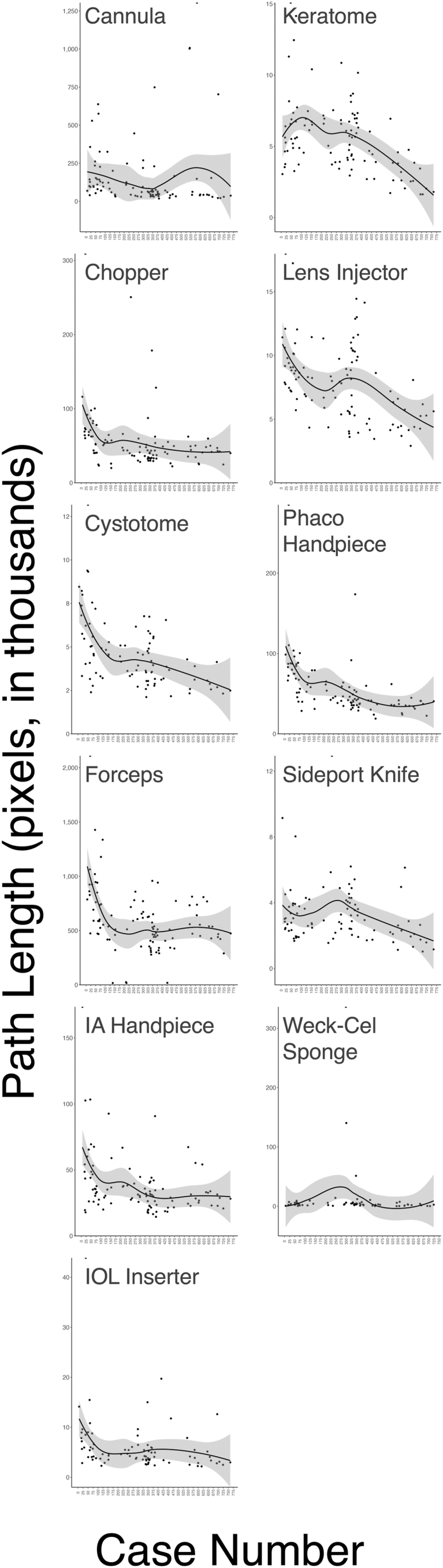
Change in total path length per case for each instrument. IA = irrigation/aspiration; IOL = intraocular lens; phaco = phacoemulsification (referring to the handpiece).

**Figure 2:**
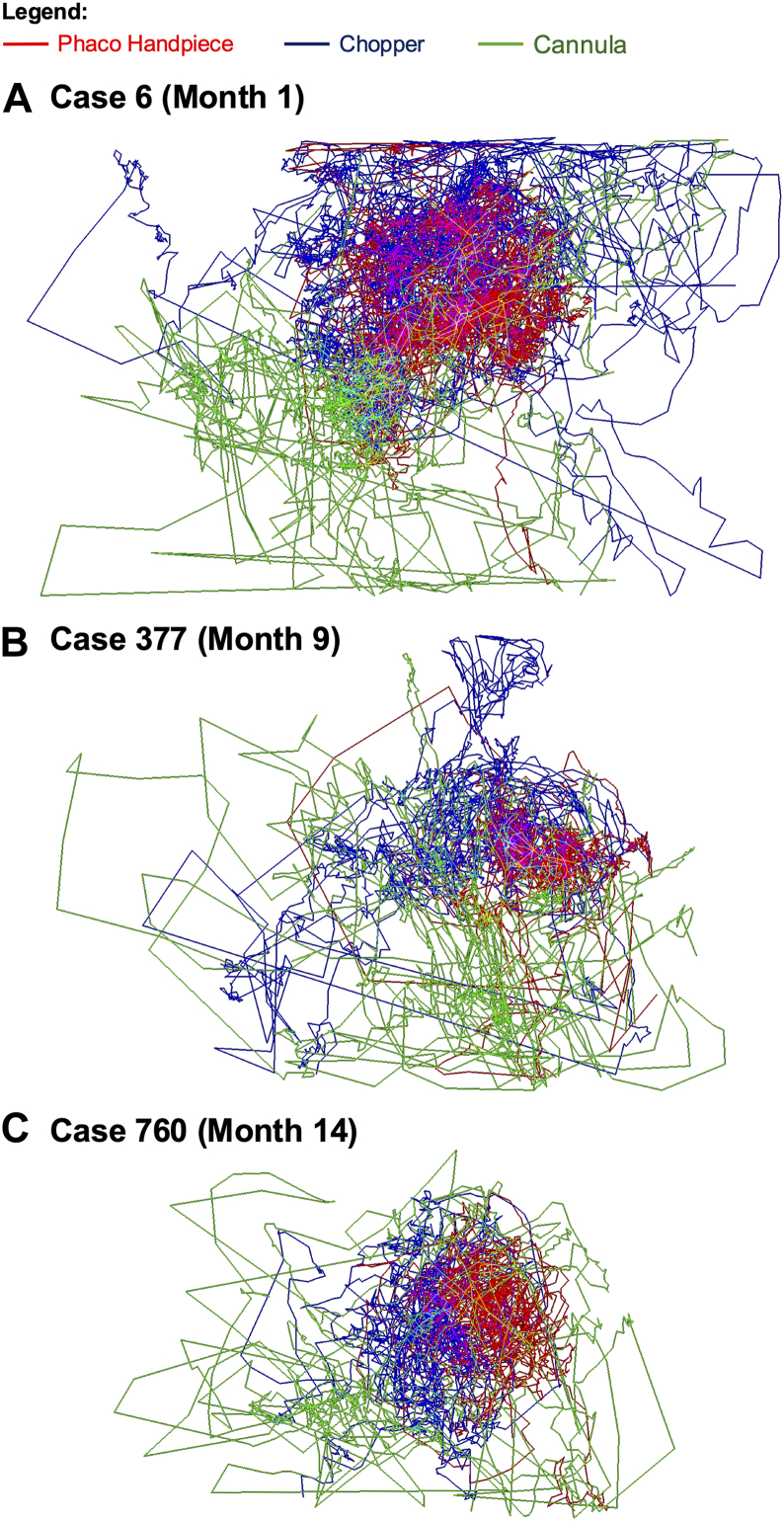
Combined motion paths for the phacoemulsification (phaco) handpiece, chopper, and cannula across surgical training. phaco = phacoemulsification (referring to the handpiece).

The data set contains 100 video recordings spanning the sixth to 760th case performed independently by a resident during their first year of surgical training (Table S1, available at www.ophthalmologyscience.org). All 11 instruments demonstrated statistically significant monotonic trends in ≥1 of the 6 motion path parameters using the Mann–Kendall test and Theil–Sen estimator with Bonferroni correction applied (Table 1). The change in total path length and average angular change per case for each instrument are presented in Figure 1 and Figure S1 (available at www.ophthalmologyscience.org), respectively. Combined motion paths for the phaco handpiece, chopper, and cannula in the first recorded case (case 6, month 1), middle (case 377, month 9), and final case (case 760, month 14) are depicted in Figure 2. Individual motion paths for all instruments at each timepoint are shown in Figure S2 (available at www.ophthalmologyscience.org).

**Table 1:** Trends in Instrument Motion Parameters

Instrument	Path Length		Velocity		Acceleration		RMS Jerk		Angular Change		Workspace Coverage	
	% Change per 10 Videos (95% CI)	Mann–Kendall *P* Value∗	% Change per 10 Videos (95% CI)	Mann–Kendall *P* Value∗	% Change per 10 Videos (95% CI)	Mann–Kendall *P* Value∗	% Change per 10 Videos (95% CI)	Mann–Kendall *P* Value∗	% Change per 10 Videos (95% CI)	Mann–Kendall *P* Value∗	% Change per 10 Videos (95% CI)	Mann–Kendall *P* Value∗
Sideport knife	**–4.0 (–7.5, –0.7)**	**0.02**	**2.4 (0.2, 0.5)**	**0.03**	–16.6 (–40.1, 3.6)	0.10	0.5 (–2.1, 3.0)	0.68	**–1.8 (–2.7, –0.9)**	**0.00**	–2.6 (–8.3, 3.3)	0.37
Keratome	**–6.2 (–8.9, –3.3)**	**0.00**	**–2.2 (–4.2, –0.4)**	**0.01**	–4.8 (–13.2, 3.3)	0.25	**–5.0 (–7.9, –2.3)**	**0.00**	**–2.1 (–2.8, –1.5)**	**0.00**	**–5.6 (–10.4, –1.3)**	**0.01**
Forceps	**–6.0 (–9.0, –3.2)**	**0.00**	0.7 (–1.3, 2.5)	0.47	34.6 (–13.1, 93.7)	0.16	**–2.2 (–4.2, –0.4)**	**0.02**	0.1 (–0.3, 0.7)	0.61	**–2.3 (–3.8, –0.8)**	**0.00**
Cannula	**–11.8 (–17.4, –6.8)**	**0.00**	**–2.8 (–5.6, –0.5)**	**0.02**	4.6 (–5.6, 16.2)	0.35	**–2.4 (–4.0, –0.8)**	**0.00**	**–1.7 (–2.2, –1.1)**	**0.00**	**–3.3 (–4.8, –2.0)**	**0.00**
Cystotome	**–8.9 (–11.8, –5.9)**	**0.00**	–0.3 (–2.2, 1.5)	0.72	0.7 (–16.4, 17.5)	0.92	–2.2 (–5.0, 0.2)	0.06	**–2.7 (–3.5, –1.8)**	**0.00**	**–7.0 (–11.5, –2.5)**	**0.00**
Phacoemulsification handpiece	**–11.5 (–14.1, –8.7)**	**0.00**	**–1.9 (–3.4, –0.5)**	**0.01**	–8.4 (–25.8, 5.7)	0.23	**–2.3 (–3.8, –1.1)**	**0.00**	–0.3 (–0.7, 0.1)	0.16	**–7.1 (–9.5, –4.9)**	**0.00**
Chopper	**–6.9 (–10.0, –4.2)**	**0.00**	0.9 (–0.5, 2.4)	0.22	–11.7 (–31.3, 8.4)	0.24	–0.2 (–1.7, 1.2)	0.70	**–1.1 (–1.5, –0.7)**	**0.00**	–0.8 (–3.0, 1.2)	0.40
IA handpiece	**–5.7 (–8.6, –2.8)**	**0.00**	**–2.5 (–3.8, –1.1)**	**0.00**	–19.6 (–54.1, 6.6)	0.16	**–3.4 (–4.7, –2.1)**	**0.00**	**–0.7 (–1.3, –0.1)**	**0.03**	**–2.3 (–4.5, –0.4)**	**0.02**
Lens injector	**–5.4 (–7.5, –3.6)**	**0.00**	**–2.9 (–4.5, –1.4)**	**0.00**	–9.7 (–28.6, 3.4)	0.16	**–4.8 (–6.8, –2.8)**	**0.00**	0.2 (–0.2, 0.7)	0.35	**–8.1 (–12.5, –4.3)**	**0.00**
IOL inserter	**–8.7 (–13.2, –4.5)**	**0.00**	**2.4 (0.1, 4.6)**	**0.046**	23.9 (–3.8, 52.8)	0.09	–1.0 (–3.1, 1.3)	0.40	**–3.0 (–3.9, –2.0)**	**0.00**	**–6.7 (–11.8, –1.0)**	**0.02**
Weck-Cel sponge	–8.9 (–18.3, 0.1)	0.052	–4.8 (–9.7, 0.3)	0.06	–15.9 (–44.6, 10.4)	0.22	–4.2 (–10.0, 1.5)	0.12	**–2.0 (–3.6, –0.3)**	**0.03**	–6.1 (–17.2, 2.9)	0.16

CI = confidence interval; IA = irrigation/aspiration; IOL = intraocular lens; RMS = root mean square.

Parameters with statistically significant trends are bolded.

∗ Bonferroni-adjusted *P* values.

All percentage change or angular change results reported as part of the trend analysis are for every 10 videos (approximately 75 consecutive cases) in this data set, not for the entire data set. Because this data set is comprised of 100 videos (754 cases), the overall change can be multiplied by 10 for each parameter. Correlation and change-point analyses were reported for the entire data set (all 100 videos, from cases 6–760).

### Incision and Entry Instruments

The keratome exhibited a 6.2% reduction in total path length (95% CI, –8.9% to –3.3%; *P* < 0.001), alongside a 2.1% (1.44°) reduction in average angular change (95% CI, –2.8% to –1.5%; *P* < 0.001). It also achieved the largest decrease in RMS jerk (motion smoothness), reducing by 5.0% (95% CI, –7.9% to –2.3%; *P* < 0.001). The sideport knife demonstrated a 4.0% reduction in path length (95% CI, –7.5% to –0.7%; *P* < 0.05) and a 1.8% (1.18°) decrease in angular change (95% CI, –2.7% to –0.9%; *P* < 0.001). It also demonstrated the highest increase in average velocity at 2.4% for every 10 videos (95% CI, 0.2%–4.5%; *P* < 0.05).

### Manipulation and Tissue Handling Instruments

The cystotome showed the greatest reduction in angular change, with a 2.7% (1.70°) decrease per 10 videos (95% CI, –3.5% to –1.8%; *P* < 0.001) and an 8.9% reduction in path length (95% CI, –11.8% to –5.9%; *P* < 0.001). The forceps exhibited a 6.0% reduction in total path length (95% CI, –9.0% to –3.2%; *P* < 0.01), with an insignificant increase in angular change of 0.1% (0.18°) (95% CI,: –0.3% to 0.7%; *P* > 0.05). The Weck-Cel sponge was the only tracked instrument that did not demonstrate a significant change in total path length (–8.9%; 95% CI, –18.3% to 0.1%; *P* > 0.05), although there was a significant reduction in average angular change by 2.0% (1.37°) (95% CI, –3.6% to –0.3%; *P* < 0.05).

### Aspiration and Irrigation Instruments

The cannula had the highest reduction in path length throughout the resident’s training, decreasing by 11.8% per 10 videos (95% CI, –17.4% to –6.8%; *P* < 0.001), with angular change reduced by 1.7% (1.16°) (95% CI, –2.2% to –1.1%; *P* < 0.001). For the IA handpiece, path length decreased by 5.7% (95% CI, –8.6% to –2.8%; *P* < 0.001), and angular change reduced by 0.7% (0.40°) (95% CI, –1.3% to –0.1%; *P* < 0.05).

### Phacoemulsification Instruments

The phaco handpiece showed the second largest reduction in path length at 11.5% (95% CI, –14.1% to –8.7%; *P* < 0.001), with an insignificant decrease in angular change by 0.3% (0.16°) (95% CI, –0.7% to 0.1%; *P* > 0.05). The phaco handpiece also had the second highest reduction in workspace coverage among instruments, with a 7.1% reduction every 10 videos (95% CI, –9.5% to –4.9%; *P* < 0.001). Throughout the resident’s training, the chopper’s path length and average angular changes were reduced by 6.9% (95% CI, –10.0% to –4.2%; *P* < 0.001) and 1.1% (0.77°) (95% CI, –1.5% to –0.7%; *P* < 0.001), respectively.

### Lens Implantation Instruments

The lens injector’s path length decreased by 5.4% (95% CI, –7.5% to –3.6%; *P* < 0.001), while there was an insignificant increase in average angular change of 0.2% (0.35°) (95% CI, –0.2% to 0.7%; *P* > 0.05). Compared with all other instruments, the lens injector also achieved the greatest reductions in workspace coverage (–8.1%; 95% CI, –12.5% to –4.3%; *P* < 0.001) and average velocity (–2.9%; 95% CI, –4.5% to –1.4%; *P* < 0.001), in addition to achieving the second largest reduction in RMS jerk (–4.8%; 95% CI, –6.8% to –2.8%; *P* < 0.001). The IOL inserter achieved an 8.7% reduction in path length (95% CI, –13.2% to –4.5%; *P*<0.01) and the second highest reduction in average angular change at –3.0% (1.68°) (95% CI, –3.9% to –2.0%; *P* < 0.001). The IOL inserter was also tied for the highest increase in average velocity per 10 videos at 2.4% (95% CI, 0.1%–4.6%; *P* < 0.05).

### Correlation Analysis

To assess how consistently motion parameters changed across the resident’s training, Spearman rank correlation coefficients (ρ) were calculated between case number and each metric (Table 2). Unlike trend estimators such as Sen slope, which capture overall magnitude and direction of change, correlation reflects the monotonicity of that change, demonstrating whether improvements occurred in a steady, consistent manner. For total path length, the phaco handpiece demonstrated the strongest negative correlation (ρ = –0.70, *P* < 0.001), consistent with its substantial reduction identified in the trend analysis. Other instruments, including the cystotome (ρ = –0.55, *P* < 0.001) and chopper (ρ = –0.45, *P* < 0.001), also showed moderate to strong correlations. In contrast, the cannula, despite exhibiting the largest reduction in total path length (11.8% per 10 videos, *P* < 0.001), had a relatively weaker correlation (ρ = –0.41, *P* < 0.001), suggesting that although the overall magnitude of improvement was high, the trajectory of change was less consistent, potentially influenced by variability across cases or learning dynamics.

**Table 2:** Correlation between Motion Path Parameters and Case Number by Instrument

Instrument	Path Length		Velocity		Acceleration		RMS Jerk		Angular Change		Workspace Coverage	
	Spearman Correlation (ρ)	*P* Value	Spearman Correlation (ρ)	*P* Value	Spearman Correlation (ρ)	*P* Value	Spearman Correlation (ρ)	*P* Value	Spearman Correlation (ρ)	*P* Value	Spearman Correlation (ρ)	*P* Value
Sideport knife	**–0.25**	**0.02**	**0.25**	**0.02**	0.19	0.07	0.04	0.73	**–0.40**	**0.00**	–0.09	0.38
Keratome	**–0.41**	**0.00**	**–0.22**	**0.03**	0.12	0.23	**–0.35**	**0.00**	**–0.53**	**0.00**	**–0.24**	**0.02**
Forceps	**–0.41**	**0.00**	0.09	0.35	–0.14	0.15	**–0.25**	**0.01**	0.06	0.55	**–0.30**	**0.00**
Cannula	**–0.41**	**0.00**	**–0.22**	**0.03**	–0.09	0.36	**–0.29**	**0.00**	**–0.42**	**0.00**	**–0.44**	**0.00**
Cystotome	**–0.55**	**0.00**	–0.04	0.71	–0.01	0.89	–0.19	0.07	**–0.54**	**0.00**	**–0.32**	**0.00**
Phacoemulsification handpiece	**–0.70**	**0.00**	**–0.26**	**0.00**	0.12	0.22	**–0.32**	**0.00**	–0.13	0.18	**–0.54**	**0.00**
Chopper	**–0.45**	**0.00**	0.13	0.19	0.12	0.24	–0.02	0.83	**–0.50**	**0.00**	–0.07	0.48
IA handpiece	**–0.39**	**0.00**	**–0.35**	**0.00**	0.15	0.14	**–0.42**	**0.00**	**–0.22**	**0.03**	**–0.24**	**0.01**
Lens injector	**–0.44**	**0.00**	**–0.35**	**0.00**	0.14	0.17	**–0.41**	**0.00**	0.11	0.29	**–0.42**	**0.00**
IOL inserter	**–0.42**	**0.00**	0.21	0.051	–0.21	0.059	–0.09	0.41	**–0.60**	**0.00**	**–0.25**	**0.02**
Weck-Cel sponge	–0.22	0.06	–0.23	0.053	0.15	0.22	–0.17	0.16	**–0.27**	**0.02**	–0.17	0.16

IA = irrigation/aspiration; IOL = intraocular lens; RMS = root mean square.

Parameters with statistically significant trends are bolded.

For average angular change, strong negative correlations were observed for the IOL inserter (ρ = –0.60, *P* < 0.001) and cystotome (ρ = –0.54, *P* < 0.001), in line with significant reductions in our trend analysis. The phaco handpiece (ρ = –0.13, *P* > 0.05), lens injector (ρ = 0.11, *P* > 0.05), and forceps (ρ = 0.06, *P* > 0.05) displayed weak, nonsignificant correlations, consistent with the absence of significant trends in angular change for these instruments.

For the other parameters such as workspace coverage, the phaco handpiece (ρ = –0.54, *P* < 0.001) and lens injector (ρ = –0.41, *P* < 0.001) demonstrated 2 of the strongest negative correlations among all instruments. For RMS jerk, a measure of smoothness, the lens injector (ρ = –0.41, *P* < 0.001) and phaco handpiece (ρ = –0.32, *P* = 0.001) also displayed some of the strongest correlations.

### Change-Point Analysis

**Figure 3:**
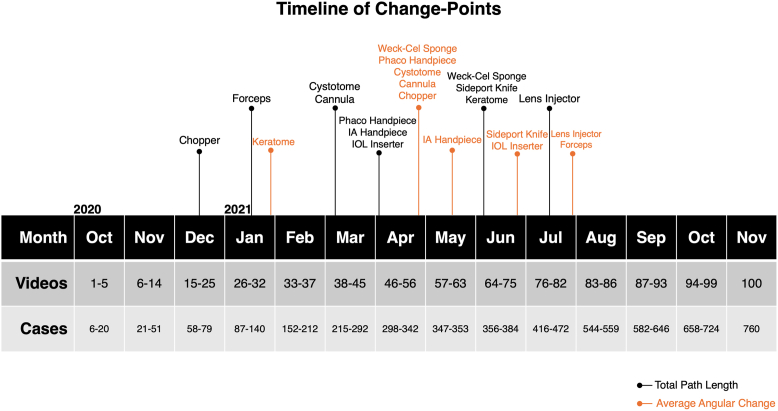
Timeline of change-points of each instrument for total path length and average angular change. IA = irrigation/aspiration; IOL = intraocular lens; phaco = phacoemulsification (referring to the handpiece).

Change-points indicating significant shifts in total path length trends were identified for all instruments using Pettitt method (Fig 3). The median change-point for total path length across all instruments occurred at case 300, aligning with a potential transition in surgical proficiency. For the sideport knife, keratome, and Weck-Cel sponge, significant change-points were observed at cases 383 (*P* < 0.005), 384 (*P* < 0.001), and 366 (*P* < 0.05), respectively. Instruments associated with manipulation, including the chopper, forceps, and cystotome, showed earlier change-points at cases 78 (*P* < 0.001), 90 (*P* < 0.001), and 235 (*P* < 0.001), respectively. Conversely, lens implantation instruments, including the lens injector and IOL inserter, displayed later change-points at cases 416 (*P* < 0.001) and 300 (*P* = 0.0051), respectively. Aspiration and irrigation instruments, including the cannula, phaco handpiece, and IA handpiece, had change-points at cases 253 (*P* < 0.001), 299 (*P* < 0.001), and 330 (*P* < 0.001), respectively, suggesting a mid-training transition.

The change-point timing of angular change differed notably from those of path length. For example, the keratome exhibited an earlier angular change-point at case 133 (*P* < 0.001), occurring 251 cases before its path length change-point. In contrast, manipulation instruments, including the chopper, cystotome, and forceps, showed delayed transitions. Angular change-points occurred at case 324 (*P* < 0.001) for the chopper (246 cases later), case 340 (*P* < 0.001) for the cystotome (105 cases later), and case 442 (*P* > 0.05) for the forceps (352 cases later).

For the remaining aspiration and irrigation instruments, as well as the lens implantation instruments, angular change-points were relatively closely aligned with path length change-points.

## Discussion

There is a growing need for objective, standardized methods of assessing surgical skill, which would reduce evaluator bias, time, and costs while being scalable across residency programs.^33^^,^^34^ With the widespread availability of surgical video data and advancements in motion tracking technology as well as artificial intelligence, operative video analysis provides a promising tool for refining surgical education.^35^^,^^36^ This study focused on the manual frame-by-frame tracking of 11 cataract surgery instruments across 6 motion path metrics, generating learning curves that benchmark key transitions in instrument use throughout a resident’s first year of surgical training.

There were significant improvements across all 6 measured parameters, highlighting distinct patterns in the learning curves of individual surgical instruments. Path length reductions indicated increasingly efficient movements, with the largest improvements seen in the cannula (–11.8%), phaco handpiece (–11.5%), and cystotome (–8.9%). The cannula’s improvements likely reflect better anticipation of fluid dynamics, whereas the phaco handpiece improvements indicate growing fluency in coordinating ultrasonic energy and foot-pedal control to ensure endothelial safety. Cystotome improvements reflect smoother capsulorhexis creation, likely due to repeated practice managing capsule elasticity and tear behavior under tension.

Reductions in angular change similarly highlighted the resident’s transition toward smoother, more deliberate motions. The IOL inserter (–3.0%) and cystotome (–2.7%) exhibited the largest reductions, reflecting the resident’s ability to carefully manipulate the IOL and maintain a continuous, circular trajectory during capsulorhexis. In contrast, the phaco handpiece (–0.3%) and lens injector (+0.2%) showed minimal differences in angular change, despite substantial reductions in path length, suggesting that for certain tasks—particularly those involving more linear, axial movements—the ability to maintain a steady, direct trajectory may matter more than frequent directional adjustments. These distinctions emphasize how surgical “skill” is a composite of diverse competencies; angle control can be critical for instruments that pivot or tear tissue, while path length reductions could be important for instruments or tasks requiring steady, targeted motions.

Reductions in RMS jerk (indicating smoother movements) and workspace coverage (indicating a more localized instrument footprint) collectively reflect maneuvers that became more controlled and precise over time, aligning with the refined movements typical of experienced surgeons. Reductions in RMS jerk were greatest for the keratome (–5.0%) and lens injector (–4.8%), while workspace coverage decreased most for the lens injector (–8.1%) and phaco handpiece (–7.1%). Velocity trends were not uniform, with an observable decrease for most instruments that favored caution and precision, but an increase for the sideport knife and the IOL inserter (each 2.4%). This may indicate growing confidence in steps that can be performed more swiftly—such as incisions—or a better sense of safe IOL insertion speed.

Our change-point analysis reinforced that learning does not follow a uniform trajectory across all surgical steps. The chopper exhibited an early shift in total path length (case 78), possibly reflecting quicker improvements in nucleus-chopping techniques. Other instruments, such as the phaco handpiece (case 299) and lens injector (416), required substantially more cases before significant efficiency gains emerged, possibly due to the higher cognitive load of foot-pedal coordination and lens placement. These results align with the high difficulty of phacoemulsification, as reflected in subjective assessments by residents and the observed rates of intraoperative complications.^37^^,^^38^ Instruments with mid-range inflection points (e.g., cannula at case 253, IA handpiece at case 330, and cystotome at case 235) suggest tasks such as fluid manipulation and capsule handling require repeated practice. Overall, the median change-point for total path length across all instruments occurred at case 300, aligning with previous analyses of the cataract surgery learning curve.^16^ In contrast, the change-points of the sideport knife (383) and keratome (384) were even later, possibly because residents continue to refine incision techniques well into their training. Moreover, angular change inflection points diverged from path length in certain instruments (e.g., relatively earlier angular refinement for the keratome by case 133), highlighting that even for the same surgical tool, different facets of skill evolve on distinct timelines. These findings suggest that measurable changes in motion patterns evolve with continued surgical experience, with an inflection point ≥300 cases, as observed for the single surgeon in this study. These findings agree with prior work that found that complication rates and efficiency improved beyond the first 80 cases and posterior capsular rupture decreased after 500 cases.^39^^,^^40^

Spearman rank correlation further underscores how different cataract surgery tasks evolve uniquely over time. The phaco handpiece had the strongest negative correlation between total path length and case number (ρ = –0.70, *P* < 0.001), indicating a consistent reduction in extraneous motion as the resident gained proficiency. The IOL inserter also had a negative correlation between case number and angular change (ρ = –0.60, *P* < 0.001). Conversely, the forceps and Weck-Cel sponge had weaker correlations, possibly because they are used in relatively straightforward tasks. Forceps largely provide countertraction, and the sponge is used primarily to absorb fluid, leaving less scope for measurable improvement. The cannula illustrates how slope-based trends can appear large (–11.8% reduction in path length per 10 videos) yet yield only moderate correlation (ρ = –0.41, *P* < 0.001). This apparent discrepancy likely arises because Spearman correlation emphasizes consistency and monotonicity in improvement, whereas Sen slope captures overall magnitude, even if changes are nonlinear. Thus, instruments with steep improvements followed by plateaus or periods of variability may demonstrate strong trend significance but weaker correlations. These findings show that skill acquisition varies across instruments, with some tasks improving steadily while others exhibit more complex, nonlinear trajectories shaped by task difficulty, learning curve dynamics, and intraoperative variability.

Motion tracking methodologies have garnered increasing attention in surgical education across multiple specialties, including laparoscopic cholecystectomy, thyroidectomy, and breast reduction, where automated analyses of instrument, hand, or eye movements have been used to objectively grade performance.^36^^,^^41^, ^42^, ^43^ Collectively, studies have included metrics such as fluidity of motion, motion economy, tissue handling, intraoperative phase duration, distance traveled, speed, and acceleration.^35^^,^^44^, ^45^, ^46^, ^47^ In ophthalmology, several studies have similarly employed motion metrics—such as total path length, movement count, and operative phase duration—to differentiate novices from experts in cataract surgery.^18^^,^^48^^,^^49^ Smith et al^18^ applied motion analysis to track instrument movements in live cataract surgeries to distinguish junior from senior surgeons, finding that expert surgeons exhibited significantly shorter total path lengths (*P* = 0.002), fewer movements (*P* = 0.05), and faster procedure times (*P* = 0.004). However, the study was limited to only 20 surgical videos, focusing on broad comparisons between 2 experience levels rather than tracking skill acquisition over time. Din et al^48^ further validated motion tracking by correlating PhacoTrack motion capture metrics with the Objective Structured Assessment of Cataract Surgical Skill, showing that experts had shorter path lengths (*P* < 0.05) and better microscope centration (*P* < 0.05) than novices, although their study was limited to 22 videos. Similarly, in a cross-sectional analysis, Balal et al^49^ analyzed 3 discrete segments of cataract surgery (capsulorhexis, phacoemulsification, and irrigation-aspiration) in 40 surgeons (20 novices, 20 experts), again demonstrating that novices had higher path lengths (*P* < 0.05), more instrument movements (*P* < 0.05), and longer times (*P* < 0.05).

To our knowledge, this is the first study to systematically track the frame-by-frame movement of essentially all fundamental cataract surgery instruments in a large data set of 100 operative videos, containing >4.5 million frames. Our results represent one of the most comprehensive labeled motion tracking data sets in ophthalmology and surgery. Building on our previous analysis of operative times in this data set, which found that the overall speed of surgery increased at a rate of 43.4 seconds for every 10 videos (95% CI, 35.1–52.7 seconds), we now integrate advanced motion path metrics to capture additional facets of technical proficiency and gain deeper insights into how residents refine their skills over time.^16^ Looking ahead, ongoing efforts aim to compare motion-based learning curves across multiple residents, semantically segment surgical instruments, and eventually develop deep learning artificial intelligence models to automate these tasks.

This study has several limitations. First, although advanced frame-by-frame motion tracking offers quantitative insights into instrument trajectories, it does not fully capture critical elements of surgical skill such as foot-pedal use, tactile feedback, bimanual dexterity, tissue manipulation forces, or 3-dimensional instrument depth. Second, this was a single-center, single-resident study conducted during the first year of surgical training; thus, our findings may not translate to different programs, more advanced trainees, or surgeons with varying learning styles. Third, we could not systematically account for case complexity (e.g., dense cataracts or zonular compromise) given the retrospective design, although it is likely that as the resident’s skills improved, more difficult cases were assigned. Later change-points in total path length for incision and entry instruments may reflect the small magnitude of change associated with these low-motion steps. Although the low variability in these measurements improves statistical power, a larger sample size may still be required to detect subtle improvements. These late inflection points are therefore more likely to represent minor refinements rather than substantial shifts in technique.

The distribution of included videos across case ranges was uneven, with a higher concentration of recordings in the early and middle phases of training. This imbalance reflects the availability of usable recordings that met our quality criteria. Moreover, the steepest improvements typically occurred in the early and middle phases of training, whereas late-stage refinements occurred more gradually; thus, wider spacing in the late phase may have facilitated detection of subtle trends. Similarly, angular change may be influenced by patient-level anatomical variability, such as axial length, anterior chamber depth, or lens thickness, which was not captured in our data. Other variations in supervisor preference, case complexity, and resident progression may have resulted in different capsulorhexis and chopping techniques. Instruments with multiple tasks were also not distinguished by task when assessing motion patterns. Heterogeneity in technique or instrument task introduces noise and would be expected to dilute monotonic findings, which would bias our findings toward the null. Despite this, we observed statistically significant, directionally consistent trends and change-points around case 300 across all instruments, supporting a true learning effect. For within-instrument insights, future studies should examine motion patterns stratified by chopping and capsulorhexis technique and distinguish specific forceps, cystotome, and cannula applications. Finally, although objective motion metrics may be promising proxies for technical proficiency, validation against clinical outcomes such as rates of capsular tear or postoperative visual acuity will be important to further confirm their relevance to patient care.

### Conclusion

This pilot study represents one of the most comprehensive operative motion path tracking data sets in ophthalmology and surgery, revealing task-specific learning trajectories. Early changes were seen in simpler, repetitive maneuvers, whereas more challenging tasks showed meaningful changes at later stages. Path length consistently decreased among all instruments, whereas velocity and angular change varied by instrument. Overall, the change-point marking a measurable difference in motion pattern in this data set occurred after 300 cataract cases had been performed independently. These findings highlight the potential of instrument-based motion tracking for feedback, benchmarking, and targeted instruction. Future investigations should aim to extend these analyses across multiple trainees and develop automated algorithms that eliminate the need for manual video annotation, enabling scalable, data-driven evaluation of cataract surgery training.
