# Supplementary material for: Quantitative Analysis of Instrument Motion Paths in Cataract Surgery across a Resident’s Training
Source: Ophthalmol Sci. 2025 Nov 26;6(2):101014. doi: 10.1016/j.xops.2025.101014 (PMC12805020; doi:10.1016/j.xops.2025.101014)
Supplement: Appendix 1 [file mmc1.pdf]

APPENDIX 1

Table of Contents

Position Coordinates and Centroid 2

Displacement and Path Length 2

Velocity 2

Acceleration 3

Jerk 3

Directional Changes 3

Workspace Coverage 4

Position Coordinates and Centroid

The annotated dataset provided frame-by-frame bounding box coordinates for each instrument. The centroid of the bounding box represents the tip of the surgical instrument. Calculating the centroid enables tracking of all instruments in space to calculate numerous parameters that quantitatively describe their motion paths. The centroid is calculated using the top-left coordinates (x_{tl}, y_{tl}) and the bottom-right coordinates (x_{br}, y_{br}) of the bounding box in each frame:

$$x_{centroid} = \frac{x_{tl}+x_{br}}{2} \qquad y_{centroid} = \frac{y_{tl}+y_{br}}{2}$$

Displacement and Path Length

Displacement (d) is a vector quantity representing the shortest straight-line distance and direction of an object from its initial to final position. We calculated displacement of each instrument tip between each pair of consecutive frames, frame i and frame $i - 1$. Displacement can indicate the efficiency of instrument usage, with smaller displacements suggesting the precise, targeted movements of an experienced surgeon:

$$d_i = \sqrt{(x_i - x_{i-1})^2 + (y_i - y_{i-1})^2}$$

Total path length (L) is the distance that the instrument tip has moved, calculated as the sum of displacement values. A longer instrument path length could indicate redundant movements such as exploratory or excessive corrective repositioning, which are more commonly observed in early-stage trainees:

$$L = \sum_{i=2}^{n} d_i$$

Velocity

Velocity (v) measures the speed of the instrument tip between frames. Consistent, moderate velocities during delicate phases of cataract surgery, such as capsulorrhexis, nucleus fragmentation, and intraocular lens (IOL) placement, indicate precise and deliberate movements. Higher velocities during phases involving continuous, repetitive, and sweeping motions (e.g., phacoemulsification or IA) may reflect efficiency and familiarity with the surgical technique. For resident ophthalmologists, adapting instrument velocity to suit each surgical task is a subtle yet important competency to optimize safety and efficiency.

Given a frame rate of 15 frames-per-second, the time (t) between frames can be calculated and used in conjunction with displacement to calculate the instantaneous velocity at frame i:

$$\Delta t = \frac{1}{15} \approx 0.0667 \qquad v_i = \frac{d_i}{\Delta t}$$

Acceleration

Acceleration (a) is the change in velocity over time, providing insights into how smoothly and steadily the instrument is handled throughout surgery:

$$a_i = \frac{v_i - v_{i-1}}{\Delta t}$$

Jerk

Jerk (j) signifies the rate of change of acceleration over time. Higher values of jerk indicate abrupt, less smooth movement. We calculated the root mean square (RMS) of all jerk values at frame i to determine an overall smoothness metric:

$$j_i = \frac{a_i - a_{i-1}}{\Delta t}$$

$$RMS_j = \sqrt{\frac{\sum {j_i}^2}{number\ of\ j_i\ values}}$$

Directional Changes

We calculated directional change metrics, which measure the number and magnitude of changes in the movement direction of the instruments. These values clarify the fluidity of motion and enable the detection of erratic angular motion which may indicate a lack of experience or confidence.

For each set of 3 consecutive positions where the instrument is present $(x_{i-2},\ y_{i-2})$, $(x_{i-1},\ y_{i-1})$, and $(x_i,\ y_i)$, we calculated the angle (θ_i) between the displacement vectors by taking the arccosine of the dot product formula:

$$\cos\theta_i = \frac{(x_i - x_{i-1})(x_{i-1} - x_{i-2}) + (y_i - y_{i-1})(y_{i-1} - y_{i-2})}{|\overrightarrow{d_i}| \cdot |\overrightarrow{d_{i-1}}|}$$

$$\theta_i = \arccos\,(cos\theta_i)$$

We calculated the number of directional changes by counting instances where $|\theta_i|$ exceeds a predefined dynamic threshold (θ_t) for each instrument:

$$\theta_t = \mu_\theta + k \times \sigma_\theta$$

where μ_θ is the mean angular change across all frames, σ_θ is the standard deviation of angular changes, and k is a multiplier, chosen as 1 for a threshold 1 standard deviation above the mean. Thus, directional changes exceeding μ_θ by a standard deviation of 1 were considered significant.

Total angular change was calculated by summing the absolute values of all θ_i:

$$\sum |\theta_i|$$

Workspace Coverage

Workspace coverage signifies the area of the workspace used by the instrument. It is derived by first collecting all the centroid positions $(x_{centroid}, y_{centroid})$ throughout the video. Using computational geometry algorithms, the convex hull can be computed by finding the smallest convex polygon containing each point. The area of the convex hull will be the workspace coverage.
