# Supplementary figures and images for: Quantitative Analysis of Instrument Motion Paths in Cataract Surgery across a Resident’s Training
Source: Ophthalmol Sci. 2025 Nov 26;6(2):101014. doi: 10.1016/j.xops.2025.101014 (PMC12805020; doi:10.1016/j.xops.2025.101014)

Average Angular Change (degrees)

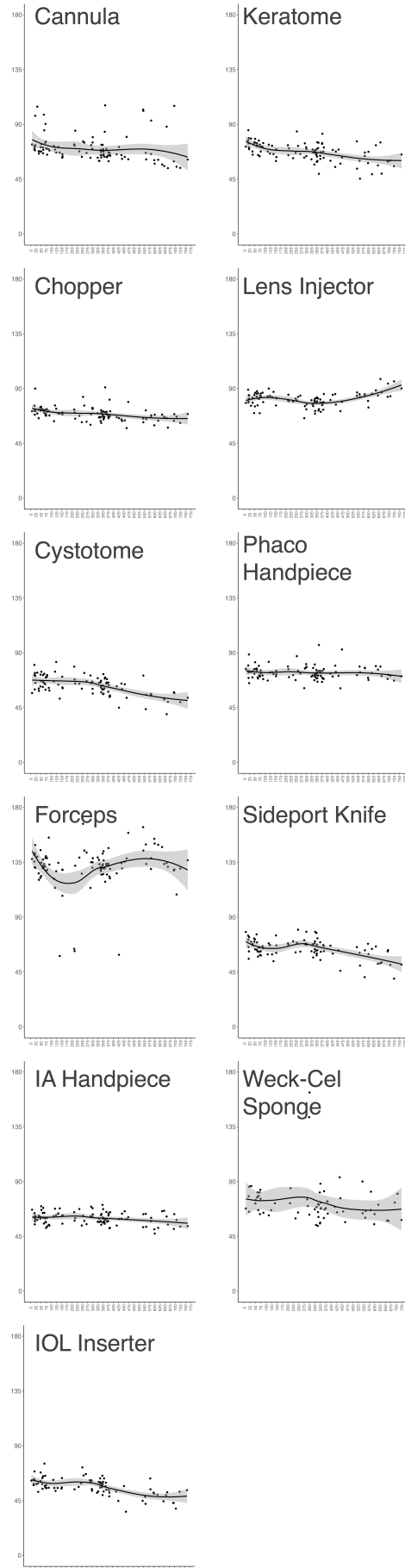

Case Number

Supplement: Supplemental_Figure_1 [file mmc2.pdf]

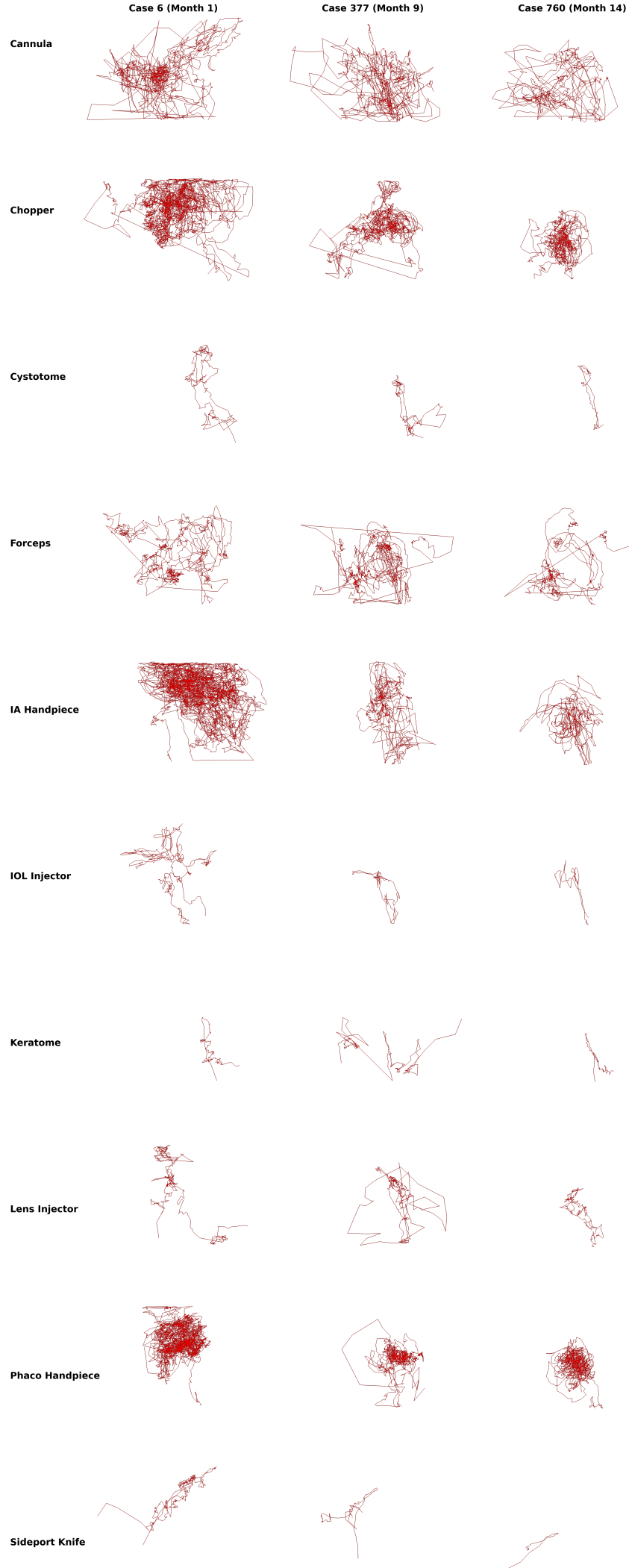

Supplement: Supplemental_Figure_2 [file mmc3.pdf]
