# Supplementary material for: Quantitative Analysis of Instrument Motion Paths in Cataract Surgery across a Resident’s Training
Source: Ophthalmol Sci. 2025 Nov 26;6(2):101014. doi: 10.1016/j.xops.2025.101014 (PMC12805020; doi:10.1016/j.xops.2025.101014)
Supplement: Supplemental_Table_1 [file mmc4.pdf]

Supplemental Table 1: Distribution of Videos by Case Range

Case Range	Number of Videos
6-50	13
51-100	14
101-150	5
151-200	3
201-250	5
251-300	8
301-350	12
351-400	15
401-450	4
451-500	3
501-550	2
551-600	6
601-650	3
651-700	4
701-750	2
751-760	1
